# Nanomaterials as protein mimics or nanologicals

**DOI:** 10.2217/nnm-2024-0064

**Published:** 2024-03-26

**Authors:** Bengt Fadeel

**Affiliations:** 1 Nanosafety & Nanomedicine Laboratory, Institute of Environmental Medicine, Karolinska Institutet, Nobels väg 13, 171 77, Stockholm, Sweden

**Keywords:** bio-corona, nanomaterials, nanomedicine, nanozymes, protein mimics

Nanotechnology is often defined as the manipulation of matter on a near-atomic scale or (sometimes) as the science and engineering of functional systems at the molecular scale. The latter definition is confusing in the sense that there is no apparent distinction between artificial systems and biological systems; life is also a nanoscale phenomenon and a living cell can be viewed as a collection of functional nanosystems [1]. I would argue, however, that confusion is fertile ground for creativity. Consider the blurring of boundaries between artificial and biological matter: is it possible for engineered nanomaterials to behave as ligands (ions/proteins) for biological receptors?

It has been noted that fullerenes, nanodiamonds, quantum dots and other ultrasmall nanoparticles approach the ‘quasi-molecular’ regime [2] and as such are more likely candidates as protein mimics [3]. Indeed, as pointed out in a recent perspective, “*the discovery of C_60_ brought the world of nanoparticles and molecules in direct contact* […] *leading to conceptual questions as to how we define a molecule* […] *and how we define a nanoparticle*” [4]. Nanoparticles have also been conceptualized as ‘mobile solids' possessing a combination of solid-state properties and dynamically changing mobility [5]. Proteins, in turn, can be classified into three main categories with respect to their function: enzymes, cell signaling/signal transduction proteins and structural or scaffolding proteins. Proteins are dynamic solids (dynamic in the sense that synthesis and degradation are a natural part of the phoenix-like life cycle of every protein), though it is noted that biomolecular condensates have emerged in recent years as an organizing principle of cellular biochemistry [6]. This raises the question whether condensates could be engineered for synthetic biology applications, but that is a topic for another day. More to the point, the question is whether nanoparticles could function as proteins, and if so, whether such ‘ancillary effects’ of nanoparticles [7] could be harnessed for therapeutic gain? In other words, could ligand-less or cargo-free nanoparticles act as ‘nanologicals’ or drugs *per se* by virtue of their intrinsic physicochemical properties or as a function of the surface-adsorbed bio-corona [8]?

Numerous studies have been published in recent years on so-called nanozymes, but the specificity of these nanozymes has been called into question [9]. Moreover, the catalytic activity of conventional nanozymes is lower than that of natural enzymes. However, single-atom engineering could potentially boost the activity of nanozymes [10]. Indeed, a recent study demonstrated that single selenium atom nanozymes with NADPH oxidase mimetic activity exhibited substantial antitumor efficacy in mice [11]. Nanomaterials may also serve as scaffolds for the regeneration of tissues [12]. Topographical features, for example, surface roughness and porosity likely play important roles, but the question is whether nanomaterials could supply biological signals in addition to biophysical cues. Specifically, do nanoparticles interact with biological receptors? There is some evidence to suggest that carbon nanotubes are sensed by the pattern recognition receptor (PRR) known as Tim4 [13], while the class B scavenger receptor SR-B1 was identified as a receptor for silica nanoparticles [14]. PRRs are tasked with sensing microorganisms as well as endogenous molecules released from damaged cells [15]. These receptors are promiscuous by design, and the aforementioned studies suggest that engineered nanomaterials can be added to the list of ligands or ‘danger’ signals. PRRs are expressed on the cell surface of immune-competent cells to detect extracellular pathogens as well as in endosomes to detect intracellular pathogens such as viruses. Recent work has demonstrated that carbon nanotubes may bind to intracellular PRRs such as GPNMB [16]. It is also worth noting that C_60_ fullerenes are antigenic; in other words, these molecules or nanostructures can trigger the production of antibodies (in mice) [17]. The same anti-C_60_ antibodies were subsequently shown to react with carbon nanotubes [18], thus providing an example of selective nanomaterial binding to a soluble immune receptor. Interestingly, chirality (i.e., left or right-handedness) of nanoparticles can influence receptor binding thereby shaping immune responses [19]. Chiral nanozymes may be useful for enantioselective catalysis, but chirality could also influence the pharmacokinetics of nanozymes, as recently shown in a mouse model of neuroinflammation [20].

To sum up, several studies in recent years have shown that nanoparticles may interact with specific biological receptors. If uncontrolled, this could lead to unwanted effects (this is known as nanotoxicity). However, the question is whether nanoparticles could be applied for therapeutic purposes, for instance, in cancer therapy? Such drug-free nanoparticles or ‘nanologicals’ could act as protein mimics, and may be more stable and cheaper to produce than natural or recombinant proteins [3]. However, if administered intravenously, a bio-corona would immediately form unless the particles are designed to avoid opsonization (it is noted that a stable and biologically meaningful bio-corona may not form on ultrasmall nanoparticles) [2]. On the other hand, the bio-corona is in principle knowable [21] and could even be exploited to direct the nanoparticles to specific tissues. If administered via inhalation or orally, other obstacles must be overcome before the nanoparticles reach their desired destination. Moreover, once the nanoparticles reach the tissues of interest, they would have to negotiate additional physiological barriers to gain access to the interior of the cell. Notwithstanding, it is conceivable that ‘nanologicals’ may find a place in the clinical arsenal. It is possible to envision different types of ‘nanologicals’ or drug-free nanoparticles: ultrasmall nanoparticles behaving as receptor-binding protein mimics, or as nanozymes (discussed above), and other nanoparticles that serve as reservoirs of biologically relevant metals. I will provide two examples of the first category here. Cui *et al.* showed that nanodiamonds could be repurposed as autophagy inhibitors to synergistically improve cancer therapy [22]. The mechanism was not disclosed, but it is possible that ultrasmall nanoparticles are perceived by cells as aggregation-prone proteins. Chen *et al.* demonstrated that metallofullerenes displayed anticancer activity in mice, but the effect could not be explained by a direct toxicity toward cancer cells *in vitro* and less than 0.05% of the dose reached the tumor *in vivo*. The authors inferred instead that the Gd-based nanoparticles boosted antitumor immune responses [23]. It will be of interest to pinpoint the receptor(s) targeted by fullerenes and nanodiamonds.

Metal-binding proteins are estimated to constitute about one third of the entire proteome. Metal-based nanoparticles could potentially act as depots of metal ions leading to increased activities of metalloproteins [24]. Moreover, iron ‘addiction’ has been identified as a particular metabolic vulnerability of cancer cells, and this therefore serves as a valid therapeutic target. Ferumoxytol (ultrasmall superparamagnetic iron oxide nanoparticles approved for the treatment of iron deficiency) showed antileukemia efficacy in cells with low expression of the iron exporting protein ferroportin and in patient-derived xenografts in mice [25]. The authors surmised that the nanoparticles triggered oxidative stress in cancer cells leading to cell death. Gao *et al.* recently devised a ferroptosis-based anticancer therapy by combining Fe_3_O_4_ nanoparticles and the targeted knockdown of iron metabolism-associated genes [26]. The approach was shown to be effective in several tumor models in mice. We evaluated Fe-doped hydroxyapatite (FeHA) as a potential means of killing cancer cells and could show that FeHA triggered ferroptosis in pancreatic ductal adenocarcinoma cells while sparing normal cells [27]. The cell death was dependent on STEAP3, a metalloreductase responsible for reducing Fe^3+^ to Fe^2+^. Ferroptosis is an iron-dependent cell death, which holds considerable promise as a therapeutic target, but other forms of metal-induced cell death including cuproptosis also merit exploration [28].

Understanding nano-bio interactions is of paramount importance for the safe implementation of engineered nanomaterials in medicine. The message that I wish to convey is that nanomaterials themselves could elicit useful biological effects; if such effects can be controlled then this could change the way in which we view nanomaterials – not merely as vehicles or excipients but as ‘nanologicals’ or drugs per se.
